# Lipid Nanoparticles in Lung Cancer Therapy

**DOI:** 10.3390/pharmaceutics16050644

**Published:** 2024-05-10

**Authors:** Hossein Omidian, Erma J. Gill, Luigi X. Cubeddu

**Affiliations:** Barry and Judy Silverman College of Pharmacy, Nova Southeastern University, Fort Lauderdale, FL 33328, USA; eg1262@mynsu.nova.edu

**Keywords:** lung cancer, lipid nanoparticles, drug delivery, drug resistance, personalized medicine

## Abstract

This manuscript explores the use of lipid nanoparticles (LNPs) in addressing the pivotal challenges of lung cancer treatment, including drug delivery inefficacy and multi-drug resistance. LNPs have significantly advanced targeted therapy by improving the precision and reducing the systemic toxicity of chemotherapeutics such as doxorubicin and paclitaxel. This manuscript details the design and benefits of various LNP systems, including solid lipid–polymer hybrids, which offer controlled release and enhanced drug encapsulation. Despite achievements in reducing tumor size and enhancing survival, challenges such as manufacturing complexity, biocompatibility, and variable clinical outcomes persist. Future directions are aimed at refining targeting capabilities, expanding combinatorial therapies, and integrating advanced manufacturing techniques to tailor treatments to individual patient profiles, thus promising to transform lung cancer therapy through interdisciplinary collaboration and regulatory innovation.

## 1. Introduction

Lung cancer remains a formidable challenge in oncology, with high mortality rates necessitating more effective therapeutic strategies. Among the critical needs is the enhancement of drug delivery systems, particularly for chemotherapeutics that often fail to target cancer cells exclusively, leading to severe side effects and reduced efficacy. The development of novel delivery mechanisms such as lipid nanoparticles (LNPs) is driven by the need to address these significant limitations.

One of the primary challenges in lung cancer treatment is overcoming drug resistance, a frequent obstacle that diminishes the effectiveness of standard treatments. Multi-drug resistance and the ability of cancer to metastasize complicate treatment protocols and highlight the necessity for innovative approaches in drug delivery systems [1,2]. The use of targeted therapy advancements, especially those that can precisely control the release and localization of drugs like doxorubicin and paclitaxel, presents a promising solution. This approach not only aims to increase the efficacy of treatment but also to reduce systemic toxicity and enhance patient outcomes [3,4,5].

Furthermore, specific issues, such as the poor prognosis in metastatic renal cell carcinoma with lung metastasis [6] and the inefficiency of radiotherapy in treating lung metastatic breast cancer [7], underline the urgent need for targeted and effective therapies. These concerns are aggravated by the limited penetration depth of traditional photodynamic therapy in treating deep-seated tumors [8], which calls for novel delivery systems that can overcome such physical and biological barriers.

Lastly, the direct targeting of lung cancer-initiating cells (CD133+ cells) with enhanced delivery systems has been identified as a critical need. This targeting is crucial for improving the prognosis and treatment outcomes in lung cancer, where current therapies often fail to eliminate these root cancer cells, leading to recurrence and metastasis [9].

In summary, the continuous evolution of drug delivery technologies, particularly through LNPs, is essential in addressing the numerous challenges in lung cancer treatment. These technologies promise to enhance the precision, efficacy, and safety of cancer therapeutics, directly responding to the unmet needs and gaps in current treatment modalities.

## 2. Lipid-Based Nanoparticles

Lipid nanoparticles, solid lipid nanoparticles (SLNs), and lipid-polymer hybrid nanoparticles (LPHNPs) represent innovative platforms in the field of drug delivery, offering unique advantages and applications in biomedicine. Lipid nanoparticles are highly regarded for their ability to control the release of therapeutic molecules, particularly those that are hydrophobic and have low bioavailability. SLNs and nanostructured lipid carriers are praised for their low toxicity, enhanced encapsulation capacity, controlled drug release kinetics, and scalability, making them pivotal in drug delivery systems [10].

Solid lipid–polymer hybrid nanoparticles (SLPNs) have emerged as a novel category, combining the beneficial properties of lipids with the stability and functional versatility of polymers. This fusion results in nanocarriers with a controlled particle size, high drug loading yield, and sustained drug release capabilities. Moreover, the hybrid structure of lipid–polymer nanoparticles improved in vitro and in vivo stability, signifying their potential as robust drug carriers [11].

**Figure 1:**
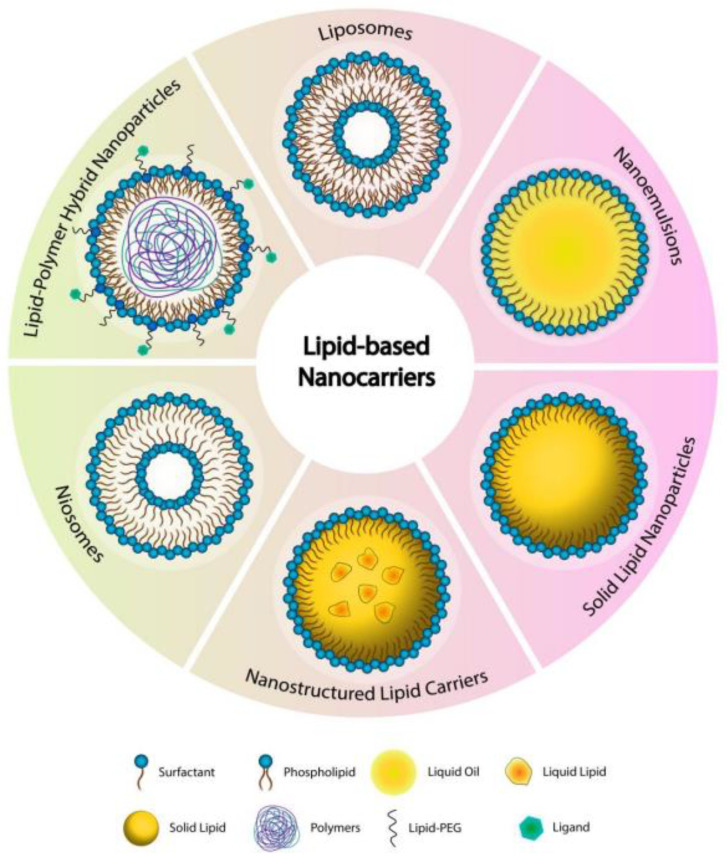
Different types of lipid-based nanocarriers [12].

The lipid-based nanocarriers are gaining significant interest by researchers working on the development of novel formulations for the pulmonary delivery of anticancer drugs owing to their biocompatible, biodegradable, non-toxic, and non-irritant nature, the ability to entrap and deliver diverse molecules in a controlled manner with enhanced bioavailability, the ability to transport across blood vessels and different membranes and barriers, in addition to the ease of preparation and scale-up. Each type of lipid-based carrier has a unique structure, as shown in Figure 1 [12].

Together, lipid-based nanoparticles, especially the advanced solid lipid–polymer hybrid types, hold significant promise for transforming drug delivery, offering targeted, controlled, and efficient therapeutic molecule transport. The continuous research and development in this area are likely to further refine these systems, overcoming current limitations and paving the way for their widespread application in treating complex diseases like cancer [13].

On the other hand, there are challenges and drawbacks associated with these nanocarriers. For instance, the structural complexity of LPHNPs can complicate their synthesis and scalability. Furthermore, the need for careful optimization of their composition and structure to ensure biological compatibility and effectiveness can be resource-intensive. Despite these challenges, the evolving synthesis methods, like microfluidics and microvortex platforms, are addressing these issues by enabling the mass production and precise size control of LPHNPs, thereby enhancing their practicality for clinical applications [14].

## 3. Chemotherapeutic Agents

Chemotherapeutic agents, fundamental to cancer pharmacotherapy, often suffer from nonspecific distribution and undesirable side effects. Lipid nanoparticles (LNPs) can be used to encapsulate these agents, enhancing their delivery directly to tumor cells while minimizing exposure to healthy tissues. Among these, microtubule inhibitors, like paclitaxel [15,16,17] and docetaxel [18,19,20,21], interfere with cell division, making them potent anticancer agents. Topoisomerase inhibitors such as etoposide [22] and 10-hydroxycamptothecin (HCPT) [23] disrupt DNA replication, essential for cancer cell proliferation. Antimetabolites, including gemcitabine [24,25] and methotrexate [26], inhibit DNA and RNA synthesis, while alkylating agents like cisplatin [27,28] directly damage DNA. Anthracyclines (e.g., doxorubicin [29,30]) and tyrosine kinase inhibitors (e.g., erlotinib [31,32,33,34]) target enzymes and signaling pathways critical for tumor growth. Additionally, diverse agents like retinoids and other unique molecules (e.g., tretinoin [35] and pazopanib [36]) play roles in modifying cellular behavior and signaling.

### 3.1. Microtubule Inhibitors

**Figure 2:**
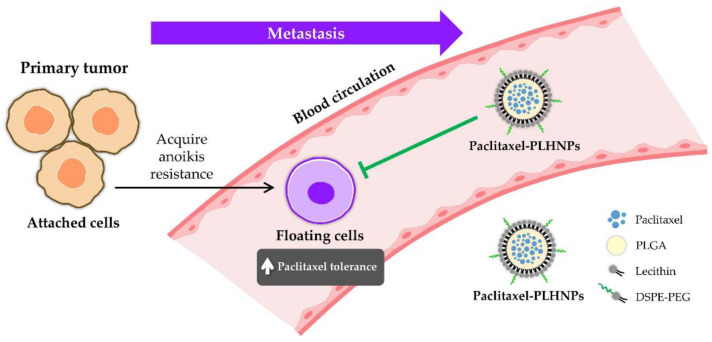
The anoikis-resistant lung cancer cells also develop drug resistance; the potential of PLGA–lipid hybrid nanoparticles (PLHNPs) to improve the efficacy of the chemotherapeutic drug paclitaxel, for eradicating anoikis-resistant lung cancer cells during metastasis [16].

**Paclitaxel**: Surface-active lipid nanovesicles containing paclitaxel were assessed for their ability to release the drug in vitro and for their cytotoxic potential, particularly focusing on their application as an aerosol for treating lung cancer. The results indicated an increase in cytotoxic potential and supported the feasibility of administering these nanovesicles as an aerosol, which could offer a new avenue for targeting lung cancer cells through inhalation therapies [15]. Building on this, poly(lactic-co-glycolic acid)–lipid hybrid nanoparticles loaded with paclitaxel were studied for their cytotoxic effects on anoikis-resistant lung cancer cells using a method that combined nanoprecipitation and self-assembly. These nanoparticles significantly diminished paclitaxel resistance in both attached and floating cancer cell states, pointing to their potential effectiveness in treating resistant forms of lung cancer, as shown in Figure 2 [16].

Further advancing this line of research, these carriers were evaluated both in vitro and in vivo on non-small cell lung cancer (NSCLC) models. The results highlighted enhanced cytotoxicity in vitro and strong anti-tumor activity in vivo, demonstrating their effectiveness as targeted therapy for lung cancer, providing a potentially more effective treatment option against resistant tumor types [17]. Additionally, RGD peptide-modified, dual-drug-loaded, redox-sensitive lipid–polymer nanoparticles containing a paclitaxel prodrug and cisplatin (RGD-ss-PTX/CDDP LPNs) were specifically developed for targeted delivery to lung cancer cells. These nanoparticles were assessed for their antitumor efficiency both in vitro and in vivo. They displayed superior antitumor efficiency and lower systemic toxicity compared to the administration of free drugs, indicating their potential as a safer and more effective lung cancer treatment option [28].

**Docetaxel**: Transitioning to another crucial drug, a nebulized lipid-based nanoemulsion loaded with docetaxel was developed for pulmonary delivery using biocompatible excipients. It was characterized aerodynamically and evaluated based on particle size, drug release, and stability. The findings revealed improved bioavailability and targeted cytotoxicity specifically towards lung carcinoma cells, suggesting its potential utility in directly delivering chemotherapy via inhalation to affected lung tissues [18]. Extending this approach, an aerosolized formulation of celecoxib encapsulated in nanostructured lipid carriers, combined with docetaxel, was tested for its anticancer efficacy in a metastatic lung cancer model. The focus was on reducing tumor size and analyzing proteomic changes. This combined treatment significantly reduced tumor growth, with the proteomic analysis supporting the effectiveness of the drug delivery system. This suggests a potentially valuable approach to treating advanced stages of lung cancer [19]. Further emphasizing targeted approaches, docetaxel and resveratrol were encapsulated in epidermal growth factor receptor-targeted lipid–polymer hybrid nanoparticles (EGF DTX/RSV LPNs) designed for the targeted treatment of non-small cell lung cancer (NSCLC). These nanoparticles were assessed for therapeutic effects both in vitro and in vivo. They demonstrated reduced systemic toxicity and enhanced tumor inhibition, marking them as a promising strategy for NSCLC treatment, leveraging targeted delivery to improve efficacy and safety [20]. Lastly, transferrin-decorated protein–lipid hybrid nanoparticles loaded with cisplatin and docetaxel (Tf-CIS/DTX-PLHN) were evaluated for their drug release behavior, cytotoxicity in vitro, and anticancer efficiency in vivo. The results showed enhanced antitumor ability both in vitro and in vivo, outperforming both single-drug-loaded nanoparticles and the free drugs. This indicates a strong potential for using targeted delivery systems to enhance the efficacy of conventional chemotherapy agents [21].

### 3.2. Topoisomerase Inhibitors

Etoposide-loaded lipid–polymer hybrid nanoparticles were optimized for targeted drug delivery using response surface methodology, and their cytotoxicity was assessed against lung cancer cell lines. These nanoparticles exhibited higher antitumor activity compared to free etoposide, suggesting their enhanced delivery and therapeutic efficacy could provide significant benefits in lung cancer treatment [22]. Continuing this theme of enhanced delivery systems, 10-hydroxycamptothecin (HCPT): PEGylated nanostructured lipid carriers loaded with 10-hydroxycamptothecin (PEG-NLCs) underwent evaluations concerning their morphology, biodistribution, cellular uptake, and in vivo anti-tumor effect against lung cancer. The results showed enhanced cellular uptake and superior efficacy against lung cancer compared to non-PEGylated forms. This indicates that PEGylation can significantly enhance the therapeutic performance of nanoparticles by improving their stability and cellular internalization [23].

### 3.3. Antimetabolites

**Gemcitabine**: Gemcitabine loaded on mannosylated solid lipid nanoparticles (Gemcitabine-SLNs) utilized mannosylation to enhance drug delivery specifically to lung cancer cells. The surface modification with mannose improved the therapeutic effectiveness and safety profile of gemcitabine in lung cancer treatment. These findings suggest that targeting specific cellular receptors through surface modifications of nanoparticles can significantly improve drug delivery and efficacy in cancer therapy [24]. Expanding on this strategy, lipid nanocapsules containing a lauroyl derivative of gemcitabine were precisely directed towards lymph nodes to address mediastinal metastases arising from lung cancer in murine models. Administered through a gel delivery system, this approach significantly prolonged survival and mitigated mediastinal metastases while notably diminishing systemic toxicity. This innovative method holds great promise for selective targeting cancer metastases while mitigating adverse effects [25]. In a similar line of targeted drug delivery, methotrexate/chitosan-coated solid lipid nanoparticles containing methotrexate (CS-SLN-MTX) were evaluated to determine the targeting efficiency of inhalation versus intravenous administration. The study examined their encapsulation efficiency and cytotoxic effects in lung carcinoma cells. Inhalation delivery led to enhanced apoptosis in lung cancer cells and achieved higher concentrations of methotrexate in the lungs, suggesting that this route could significantly improve the therapeutic outcomes by maximizing local drug concentration at the tumor site [26].

### 3.4. Alkylating Agents

These chemicals add alkyl groups to the DNA molecule, which can result in significant DNA damage and prevent the cancer cells from replicating their DNA and dividing. Transitioning into alkylating agents, cisplatin/ultra-small lipid–polymer nanoparticles loaded with cis-platinum (USLPNPs-CDDP) were synthesized using a one-pot method to address lung cancer therapy, with a focus on overcoming drug resistance in A549/CDDP cells. These nanoparticles showed enhanced anticancer benefits and the ability to bypass drug resistance mechanisms compared to free cis-platinum, suggesting a promising advancement in treating resistant forms of lung cancer [27].

### 3.5. Anthracyclines:

**Doxorubicin**: Post-bombesin-decorated nanostructured lipid carriers loaded with doxorubicin and DNA were studied to compare their targeting efficiency for lung cancer cells before and after bombesin decoration. The evaluation focused on cell targeting and nuclear delivery efficiencies. The results indicated superior transfection efficiency and tumor inhibition with post-bombesin decoration compared to their pre-decoration counterparts, highlighting the importance of targeted delivery modifications for improved cancer treatment efficacy [29]. Expanding on this concept, transferrin-conjugated, doxorubicin-loaded, lipid-coated poly d,l-lactic-co-glycolic acid nanoparticles (TF-LPs) were developed as targeted vectors for lung cancer therapy. These nanoparticles underwent evaluations for particle size, zeta potential, encapsulation efficiency, and drug release characteristics. The results indicated enhanced tumor growth inhibition and selective cytotoxicity towards lung carcinoma cells, demonstrating the potential of transferrin-conjugated carriers in improving the delivery and efficacy of chemotherapeutic agents in lung cancer treatment [30].

### 3.6. Tyrosine Kinase Inhibitors

These medications block the action of enzymes known as tyrosine kinases, which are involved in many cell functions, including signaling pathways that control cell growth and division. By blocking these signals, these inhibitors can slow down or stop the growth of cancer cells, which are as follows:

**Erlotinib**: Core-shell lipid–polymer hybrid nanoparticles (CSLPHNPs) loaded with erlotinib were engineered with a polymeric core enveloped by a lipid shell, tailored for the targeted delivery of erlotinib. The assessment of their physicochemical characteristics, stability, and cellular uptake demonstrated superior uptake and efficacy of erlotinib in lung cancer cells compared to conventional erlotinib solutions [31]. Furthermore, utilizing localized delivery, microparticles containing erlotinib-loaded solid lipid nanoparticles (ETB-SLN DPI) were formulated for local delivery to the lung using a dry powder inhaler. Their cytotoxicity and aerodynamic properties were assessed in NSCLC cells, showing enhanced cytotoxicity and improved delivery to lung tissues. This indicates a potential for effective NSCLC treatment, leveraging localized drug delivery to enhance drug concentration at the site of the tumor and reducing systemic side effects [32]. Building on this approach, polyethylene glycol (PEG)ylated polypeptide lipid nano capsules loaded with erlotinib were developed with a core-shell structure to enhance the anticancer efficacy of erlotinib, featuring pH-responsive release in acidic tumor environments. The results showed superior tumor regression and decreased tumor volumes in non-small cell lung cancer models [33]. Furthermore, erlotinib was also loaded into both liposomes and nanostructured lipid carriers for a comparative in vitro evaluation against the A549 lung cancer cell line, focusing on encapsulation efficiency, particle size, and anticancer activity. The nanostructured lipid carriers exhibited higher encapsulation efficiency, stability, and induced greater cell apoptosis compared to liposomes [34].

**Afatinib**: Transferrin-modified redox-sensitive lipid–polymer hybrid nanoparticles loaded with afatinib (Tf-SS-Afa-LPNs) were rigorously examined for their therapeutic effects both in vitro and in vivo, with a specific focus on drug release behavior in the presence of glutathione and pharmacokinetics. These nanoparticles demonstrated enhanced drug release and increased plasma concentrations, resulting in remarkable tumor volume inhibition. The targeted delivery and redox-sensitive release mechanism provided a significant advantage in treating cancer effectively, showcasing their potential in enhancing the pharmacological profile of afatinib [37].

**Gefitinib**: Gefitinib-loaded nanostructured lipid carriers (GEF-NLC) were optimized using a design of experiments approach for lymphatic delivery to treat metastatic lung cancer. The evaluation of their physicochemical properties and cytotoxicity revealed enhanced gefitinib delivery with controlled release and reduced systemic toxicity, suggesting an improved therapeutic strategy for lung cancer treatment [38]. Furthermore, biodegradable lipid nanoparticles loaded with gefitinib and azacitidine (GEF-AZT-NLC) were developed to co-deliver these drugs for treating metastatic-resistant lung cancer. The comprehensive characterization of these nanoparticles included physicochemical properties, drug release, cytotoxic activity, and hemocompatibility evaluation. The results showed promise in treating resistant lung cancer cells while demonstrating hemocompatibility and safety, indicating their potential effectiveness in clinical settings [39].

**Crizotinib**: Crizotinib-loaded lipid–polymer hybrid nanoparticles (CL-LPHNPs) were optimized using the Box–Behnken design involving variables such as polymer amount, soy phosphatidylcholine, and 1,2-Distearoyl-sn-Glycero-3-Phosphoethanolamine-Poly (ethylene glycol) (DSPE-PEG). The evaluation of drug release, cellular uptake, and gene expression in lung cancer cells indicated enhanced drug release and a significant reduction in cell viability and key oncogenic pathways. These findings suggest that the optimized formulation could offer a more effective approach for targeting lung cancer cells and reducing tumor viability [40].

**Sunitinib**: Biotin-decorated sunitinib-loaded nanostructured lipid carriers (biotin-SUN-NLCs) were developed and optimized for the targeted chemotherapy of lung cancer. These carriers were evaluated for their cellular uptake and cytotoxicity. The results demonstrated a significant enhancement in cytotoxicity and cellular uptake, suggesting that biotin decoration on nanostructured lipid carriers can effectively target and deliver chemotherapeutic agents to cancer cells, thereby enhancing the therapeutic outcomes in lung cancer treatment [41].

### 3.7. Retinoids

These vitamin A derivatives regulate cell differentiation and growth. In cancer therapy, they are used to induce the differentiation and reduce the proliferation of cancer cells, thereby controlling the progression of the disease.

**Tretinoin:** Tretinoin-loaded lipid-core nanocapsules (TTN-LNC) were specifically evaluated for their effects on apoptosis and cell cycle arrest in A549 cells that are resistant to tretinoin. The study also analyzed gene expression related to the cell cycle. The results indicated that these nanocapsules successfully overcame drug resistance, induced apoptosis, and arrested the cell cycle in lung adenocarcinoma cells. These findings suggest that TTN-LNC could be a viable strategy to counteract resistance and enhance the therapeutic efficacy of tretinoin in treating lung cancer [35]. Expanding upon the theme of retinoids, all-trans retinoic acid (ATRA): a liposome formulation incorporating dioleoyl trimethylammonium propane (DOTAP) and cholesterol, encapsulating all-trans retinoic acid (ATRA), was developed with a focus on creating a pH-responsive liposome to enhance the delivery of ATRA to lung cancer cells. This formulation aimed to improve the intracellular drug accumulation and increase the anticancer effect on A549 cells. The enhanced delivery of ATRA to lung cancer cells led to significantly increased cell death compared to free ATRA, suggesting that this pH-responsive liposome could effectively improve the delivery and therapeutic impact of ATRA in lung cancer treatment [42]. Furthering the innovation in retinoid-based treatments, hybrid lipid nanocore–protein shell composites loaded with genistein (GNS) and all-trans retinoic acid (ATRA) were developed for inhalable lung cancer therapy. These composites were designed for targeted delivery and enhanced cellular uptake, and their therapeutic efficacy and deep lung deposition were evaluated in lung carcinoma-bearing mice. The study concluded that these composites had superior efficacy against lung carcinoma and favorable aerosolization characteristics, making them highly effective for targeted therapy [43].

### 3.8. Miscellaneous/Unique Mechanisms

**Pazopanib**: Pazopanib-loaded solid lipid nanoparticles (Pazo-SLNs) encapsulated pazopanib in an amorphous form within solid lipid nanoparticles. The evaluation of these nanoparticles focused on particle size, entrapment efficiency, and drug release characteristics. The results indicated enhanced oral bioavailability and therapeutic efficacy against non-small cell lung cancer (NSCLC), demonstrating that solid lipid nanoparticles can significantly improve the delivery and effectiveness of pazopanib in treating this challenging cancer type [36].

**Phospho-sulindac (OXT-328)**: A solid lipid nanoparticle-encapsulated phospho-sulindac (SLN-PS) was evaluated for its antitumor activity both in vitro and in xenograft models, with a focus on pharmacokinetics and mitochondrial targeting in mice. The results showed that SLN-PS significantly suppressed the growth of lung cancer xenografts and induced oxidative stress in the tumors. This indicates that encapsulating phospho-sulindac in solid lipid nanoparticles enhances its delivery and antitumor activity, offering a promising approach for lung cancer therapy [44].

**Lipid-Modified Platinum Derivatives**: Epidermal growth factor receptor-targeted chitosan nanoparticles have been crafted for the delivery of lipid-modified platinum derivatives, utilizing a combinatorial design to optimize the delivery system for hydrophobic drugs. This approach has significantly improved the cytotoxicity of cisplatin in both platinum-sensitive and -resistant lung cancer cells. The targeted nanoparticles ensure enhanced cellular uptake and efficacy, representing a significant advancement in overcoming drug resistance in lung cancer therapies [45].

Table 1 provides a detailed summary of various lipid-based nanocarrier systems used for the encapsulation and delivery of different active ingredients, targeting a range of therapeutic mechanisms in cancer treatment. This compilation highlights the diversity in design and application of lipid nanotechnologies aimed at enhancing efficacy and specificity of cancer therapies.

## 4. Gene Therapy

Gene therapy represents a transformative avenue for cancer treatment, leveraging LNPs to deliver genetic material directly to cells. DNA-based therapies such as plasmid DNA [46,47] and RNA-based strategies including siRNA [48,49,50,51,52,53,54,55,56] and siRNA and DNA therapies [57] offer mechanisms to silence or modify gene expression crucial to cancer progression. These approaches aim to rectify genetic errors or inhibit oncogenic pathways at the molecular level.

### 4.1. DNA-Based Therapies

**Plasmid DNA therapies:** Cationic solid lipid nanoparticles designed for the delivery of the *p53* gene to lung cancer cells include components such as tricaprin, 3β-[*N*-(*N*′,*N*′-dimethyl aminoethane) carbamoyl] cholesterol, dioleoyl phosphatidylethanolamine, and Tween 80. This formulation was specifically assessed for gene transfer, expression, and its ability to induce apoptosis. The results indicated the efficient induction of apoptosis and tumor growth inhibition in lung cancer cells, underscoring the potential of these nanoparticles as a powerful tool for gene therapy aimed at restoring tumor suppressor function in cancer cells [46]. Building on this approach, transferrin-modified solid lipid nanoparticles co-encapsulated with doxorubicin and plasmid DNA (T-SLN/DE) were formulated to facilitate both gene and drug co-delivery to lung cancer cells. The particles were characterized by their size, surface charge, transfection efficiency, and cytotoxicity. The combination of gene therapy and chemotherapy delivered by these nanoparticles showed a remarkable therapeutic effect, suggesting a powerful approach for treating lung cancer by integrating multiple therapeutic modalities [47].

### 4.2. RNA-Based Therapies

**siRNA and DNA therapies:** The development of pH-sensitive lipid-based nanostructured lipid carriers that incorporate the GALA peptide has markedly advanced targeted therapies in lung cancer treatment. These carriers utilize the GALA peptide for targeting and have significantly enhanced the gene silencing capabilities of siRNA within the lung endothelium. The research has focused on understanding how these carriers facilitate the endosomal escape and selectivity towards specific organs. The results demonstrate robust lung endothelium gene knockdown and significant control over lung metastasis, indicating a promising approach for targeting and effectively managing lung-related conditions [48]. Additionally, transferrin-coated nanostructured lipid carriers loaded with plasmid DNA (Tf-NLC/pEGFP) were developed to enhance targeted gene delivery to lung cancer cells. Evaluations of transfection efficiency and gene loading both in vitro and in vivo demonstrated significantly higher transfection efficiency, highlighting their potential for lung cancer gene therapy. This targeted delivery system leverages the natural biology of cancer cells to enhance the uptake of therapeutic genes, potentially improving treatment outcomes [49].

**Figure 3:**
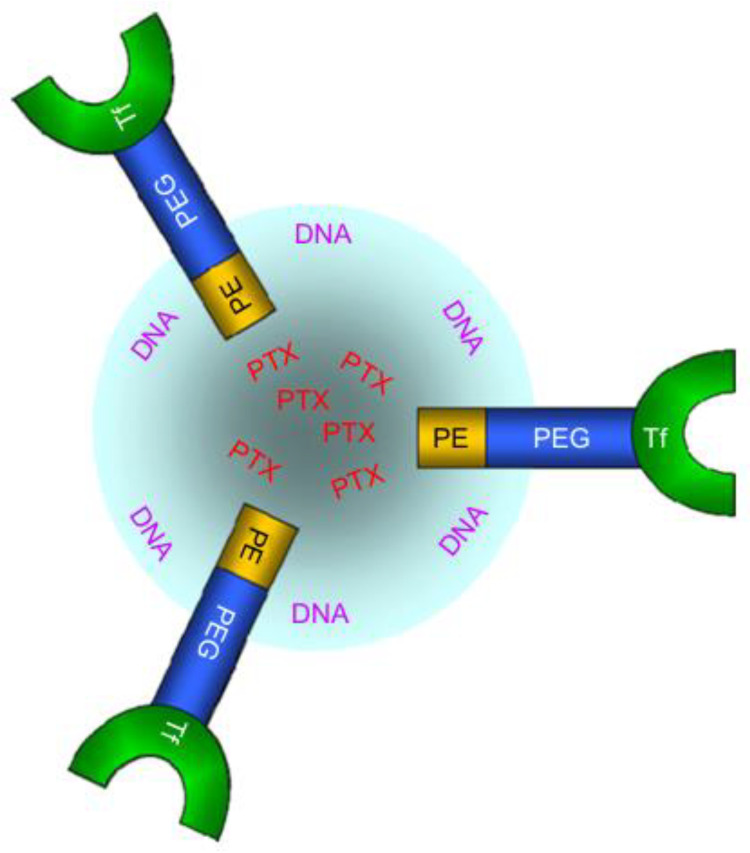
A schematic diagram of Tf-PTX-DNA-NLC (transferrin-decorated nanostructured lipid carriers co-encapsulating paclitaxel and DNA) [57].

Delving deeper into multifunctional carriers, transferrin-decorated nanostructured lipid carriers were designed to co-encapsulate paclitaxel and DNA (Tf-PTX-DNA-NLC), aiming for dual drug and gene delivery to lung cancer cells. The fabrication of Tf-PTX-DNA-NLC involved a sequential process: initially, PTX-DNA NLC formation, followed by the preparation of Tf-Peg-Pe (transferrin-conjugated polyethylene glycol-phosphatidylethanolamine), which was subsequently applied to the surface of PTX DNA-NLC, resulting in the formation of Tf-PTX-DNA-NLC, as depicted in Figure 3. These carriers were evaluated for their size, zeta potential, and transfection efficiency both in vitro and in vivo. The results showed enhanced antitumor activity and gene transfection efficiency, with improved targeting to lung cancer cells. Such dual-function carriers represent a significant advancement in cancer therapy, combining the cytotoxic effects of chemotherapy with the corrective potential of gene therapy to treat lung cancer more effectively [57].

Building on the concept of targeted gene silencing, cationic solid lipid nanoparticles containing plasmid DNA designed for STAT3 downregulation (cSLN/plasmid DNA complexes) were developed to deliver RNA interference molecules targeting STAT3 in resistant lung cancer cells. The evaluations included nanoparticle size, protection against degradation, and gene expression. These nanoparticles significantly reduced STAT3 expression, thereby increasing the sensitivity of these cancer cells to chemotherapy. This approach shows promise in overcoming resistance mechanisms in lung cancer, potentially leading to more effective therapeutic strategies [50].

Tripeptide lipid nanoparticles were developed using tripeptide lipids, sucrose laurate, and folate-PEG(2000)-DSPE to co-deliver paclitaxel and anti-VEGF siRNA, aimed at enhancing anti-tumor activity in lung cancer. These nanoparticles underwent evaluations for cellular uptake, drug release, in vitro efficacy, and in vivo tumor inhibition. The results demonstrated potent anti-tumor activity and reduced toxicity, suggesting that these nanoparticles hold promise for enhancing the efficacy of lung cancer therapy by simultaneously targeting the tumor microenvironment and the cancer cells themselves [51].

Concluding with the integration of chemotherapy and gene therapy, self-assembled lipid nanoparticles designed for the co-delivery of cisplatin and siRNA targeting endonuclease xeroderma pigmentosum group F (XPF)(siRNA-XPF/cisplatin-LNPs) aimed to enhance antitumor activity and overcome resistance. These nanoparticles were evaluated for drug and gene delivery efficiency and cytotoxicity. The treatment enhanced the expression of apoptosis markers and improved cytotoxicity in cisplatin-resistant lung cancer cells, demonstrating the potential of combining chemotherapy with gene therapy to tackle drug resistance effectively [52].

Cationic liposomes were generated using a lipid-film-coated proliposome method and were utilized to deliver CYP1A1 siRNA, aimed at silencing the CYP1A1 gene in human alveolar adenocarcinoma cell lines and BALB/c nude xenografts. The study assessed gene expression, apoptosis, and tumor inhibition. The silencing of the CYP1A1 gene led to significant tumor growth inhibition and decreased enzymatic activity associated with the gene, illustrating the potential of targeted gene therapy to disrupt cancer cell growth and metabolism effectively [54].

Finally, a lipid nanoparticle (LNP) encapsulating an siRNA inhibitor of glutathione-s-transferase P (GSTP) was developed for advanced non-small cell lung cancer (NSCLC) treatment, with a particular focus on Kirsten rat sarcoma virus (KRAS) mutant tumors. The efficacy and safety of this treatment, branded as NBF-006, were explored through in vitro studies, in vivo pharmacokinetics, immunological assessments, and clinical safety in Phase 1 trials. NBF-006 was well-tolerated and displayed a significant potential for treating KRAS mutant tumors, showing promising safety and efficacy profiles that support further clinical development [55]. Similarly, another lipid nanoparticle (LNP) encapsulating glutathione S-transferase P (GSTP) siRNA targeting Kirsten rat sarcoma virus (KRAS)-driven non-small cell lung cancer (NSCLC) was investigated for its biodistribution, antitumor activities in Kirsten rat sarcoma virus (KRAS) mutant NSCLC xenograft models, and survival in orthotopic lung tumor models. This treatment led to significant tumor growth inhibition, prolonged survival, and was well tolerated in the preclinical efficacy models, highlighting its potential as an effective therapeutic strategy in targeting specific genetic drivers of lung cancer, which are often associated with poor prognosis and limited treatment options [56].

**Anti-miRNA:** Solid lipid nanoparticles loaded with anti-microRNA oligonucleotides targeting microRNA-21 (AMO-CLOSs) have been rigorously studied for their cellular uptake, antisense efficiency, and impact on the proliferation, migration, and invasion of tumor cells. These nanoparticles exhibited high antisense efficiency, effectively reducing tumor cell proliferation, migration, and invasion. The results underline the potential of using targeted anti-microRNA strategies within solid lipid nanoparticles to precisely inhibit specific microRNAs like microRNA-21, which is known to play a crucial role in the progression of various cancers, including lung cancer [58]. Building on this concept, quaternary and tertiary amine combination lipid nanoparticles (QTsome) loaded with antimiR-21 were developed specifically for the therapeutic delivery of antimiR-21, targeting miR-21 in lung cancer cells. These nanoparticles were assessed for their in vitro effectiveness and in vivo tumor regression in a xenograft mouse model. The QTsome demonstrated effective miR-21 inhibition, resulting in tumor regression and increased sensitivity to chemotherapy. This approach highlights the therapeutic potential of modulating miR-21 as a strategy to enhance the efficacy of conventional chemotherapy in lung cancer treatment [59].

**miRNA therapy:** Coated cationic lipid nanoparticles entrapping microRNA-660 (CCL660) were administered intraperitoneally in SCID mice carrying lung cancer patient-derived xenografts (PDXs). The study focused on analyzing the inhibition of tumor growth. The findings revealed a significant reduction in tumor growth and inhibited cancer cell proliferation, demonstrating the efficacy of targeted microRNA delivery via lipid nanoparticles to suppress tumor growth effectively in a highly relevant clinical model [60].

**Antisense oligonucleotides:** Lipid nanoparticles loaded with a gapmer antisense oligonucleotide against Bcl-2 (G3139-GAP) were developed to deliver a modified antisense oligonucleotide targeting the *Bcl-2* gene, crucial for lung cancer treatment. The efficacy of these nanoparticles was assessed both in vitro and in a murine xenograft model. The results showed significant tumor growth inhibition and prolongation of survival in mice, underscoring the effectiveness of targeting Bcl-2 as a therapeutic strategy and highlighting the potential of lipid nanoparticles to enhance the delivery and efficacy of antisense oligonucleotides [61].

Table 2 summarizes the various lipid-based nanoparticle systems designed for the targeted delivery of genetic material and chemotherapeutic agents in cancer treatment. The table highlights innovative strategies such as gene therapy, RNA interference, and combination therapies aimed at enhancing tumor targeting and overcoming drug resistance in lung cancer and other malignancies.

## 5. Natural-Based Treatments

Exploring compounds derived from nature, such as polyphenols and terpenoids, introduces potential anticancer properties with lower toxicity profiles. Curcumin [62,63,64,65,66] and other polyphenols exhibit anti-inflammatory and anticancer effects. Terpenoids like oridonin [67,68] and other natural extracts have shown efficacy against various cancer mechanisms. LNPs can enhance the bioavailability and targeted delivery of these naturally occurring compounds, potentially overcoming the limitations of traditional administration routes.

### 5.1. Polyphenols

**Curcumin**: Catanionic lipid nanosystems incorporating curcumin were evaluated for their pharmacokinetics and anti-lung cancer activity, with a specific focus on comparing their bioavailability and cytotoxic effects to those of free curcumin. The study demonstrated that these nanosystems significantly enhanced anti-lung cancer activity and effectively induced apoptosis in Lewis lung carcinoma (LLC) cells. The results suggested that catanionic lipid nanosystems can improve the delivery and therapeutic efficacy of curcumin, offering a potential advantage over traditional forms of this naturally occurring compound [62]. Building on the potential of curcumin, curcumin-loaded solid lipid nanoparticles (Cur-SLNs) were specifically optimized for the treatment of lung cancer. The assessment of these nanoparticles included particle size, drug release, and in vitro cytotoxicity. The findings indicated that these Cur-SLNs exhibited higher cytotoxicity against lung cancer cells compared to free curcumin, with significant uptake by the cells. This suggests that the solid lipid nanoparticles enhance the delivery and efficacy of curcumin, making it more effective in targeting and killing cancer cells [63]. Further exploring the applications of curcumin, solid lipid nanoparticles loaded with curcumin were characterized for their physicochemical properties and analyzed for anticancer efficiency both in vitro and in vivo, with a particular emphasis on apoptosis induction. These nanoparticles showed increased targeting to lung and tumor tissues, significantly enhancing tumor inhibition efficiency. This improvement points to the potential of solid lipid nanoparticles to serve as an effective vehicle for curcumin, optimizing its therapeutic benefits in lung cancer treatment [64].

**Genistein**: Multicompartmental lipid–protein nanohybrids (MLPNs) were designed for the combined delivery of tretinoin (TRE) and genistein (GEN), targeting lung cancer. These nanohybrids were evaluated for their cytotoxic effects against A549 cancer cells and assessed for apoptotic activation in lung-cancer-bearing mice. The MLPNs exhibited enhanced cytotoxicity and significant apoptotic activation in the treated mice, indicating their potential as a promising approach for lung cancer therapy [69].

**Silymarin**: Silymarin loaded into solid lipid nanoparticles (SLNs) was investigated for its cytotoxic and apoptotic effects on lung and breast cancer cells, comparing the efficacy of silymarin alone and when encapsulated in SLNs. The study revealed that silymarin delivered via SLNs induced higher rates of apoptosis and significantly reduced cell growth compared to silymarin administered alone. This suggests that the encapsulation in solid lipid nanoparticles enhances the delivery and efficacy of silymarin, making it a more potent option for cancer treatment [70].

**Apigenin**: Hyaluronic acid-based nanostructured lipid carriers encapsulating apigenin were developed with the aim of inhibiting Nrf2 in conjunction with docetaxel in lung cancer cells. The targeted delivery of apigenin was evaluated for its potential to enhance the chemotherapeutic effect against non-small cell lung cancer (NSCLC), particularly focusing on overcoming resistance to docetaxel. The studies showed increased cytotoxicity and induction of apoptosis in lung cancer cells, indicating significant potential for this combination to overcome drug resistance, thereby improving treatment outcomes in NSCLC [71].

**Phyllanthi Tannin**: Phyllanthus emblica (commonly known as Indian gooseberry) tannins loaded into solid lipid nanoparticles (PTF-SLNs) and were prepared and characterized specifically for treating lung cancer. The pharmacodynamics and safety of these nanoparticles were evaluated on tumor-bearing mice. The findings demonstrated strong anti-tumor efficacy, lower IC50 values indicating higher potency, and reduced organ damage compared to other treatments. This suggests that PTF-SLNs could be a viable and effective option for lung cancer therapy, providing potent anticancer effects while minimizing harm to normal tissues [72].

### 5.2. Terpenoids and Terpenes

**Oridonin**: Anisamide-lipid calcium phosphate nanoparticles loaded with oridonin (AS-ORD LCPs) were evaluated for their particle size, release behavior, stability, pharmacokinetics, and tumor-targeting ability in vivo. The study showed that these nanoparticles provided sustained release, improved tumor targeting, and increased anti-tumor efficiency in mice. These findings underline the potential of using anisamide as a targeting ligand in lipid calcium phosphate nanoparticles to enhance the delivery and efficacy of oridonin, suggesting a promising approach for cancer therapy [67]. Expanding on this therapeutic strategy, lipid-layered nanoparticles co-encapsulating cisplatin and oridonin (D/O-NPs) were specifically prepared to deliver both cisplatin and oridonin together for synergistic lung cancer therapy. These nanoparticles underwent rigorous evaluations for their cytotoxic effects in vitro and their ability to inhibit tumors in vivo within a lung cancer model. The results demonstrated enhanced cell toxicity in vitro and significant antitumor effects in vivo, particularly against a resistant lung cancer model. This suggests that the combined delivery of these two agents can significantly improve treatment efficacy by leveraging their synergistic effects [68].

**Geraniol**: Geraniol encapsulated in nanostructured lipid carriers (NLC) was studied to improve its stability and bioavailability. The evaluation focused on its effects on cell viability and mitochondrial function. The results demonstrated enhanced cytotoxicity in A549 lung cancer cells, with significant increases in cell death and inhibition of cell migration. These findings highlighted the potential of NLCs to enhance the therapeutic properties of geraniol, making it a more effective agent against lung cancer [73].

**Triptolide**: Continuing with the theme of potent terpenoids, lipid–polymer hybrid nanoparticles encapsulating both paclitaxel and triptolide (P/T-LPNs) were developed for simultaneous delivery to lung cancer cells in a mouse model, aiming to enhance the therapeutic effects and minimize drug resistance. These nanoparticles demonstrated greater cytotoxicity and significant reductions in tumor volume in the mice, suggesting their superiority over treatments involving either drug alone [74].

**Beta-Elemene**: Further exploring synergistic combinations, doxorubicin and beta-elemene co-loaded, pH-sensitive nanostructured lipid carriers (DOX/ELE Hyd NLCs) were developed for the co-delivery of these drugs, designed to exploit the acidic environment of tumors. The evaluation of these carriers focused on their synergistic effects and tumor inhibition capabilities in lung cancer models. The findings demonstrated significantly enhanced cytotoxicity and tumor inhibition, indicating a synergistic anticancer effect that could offer an advanced strategy for lung cancer therapy [75].

**Sclareol**: Lastly, sclareol-loaded solid lipid nanoparticles were tested for their genotoxicity and cytotoxicity on lung cancer cells, including assessments of particle size, encapsulation efficiency, and drug release. The results indicated that these nanoparticles induced apoptosis and inhibited the growth of A549 lung cancer cells. This efficacy suggests that sclareol, when delivered through solid lipid nanoparticles, could provide a viable therapeutic option for treating lung cancer by effectively targeting and killing cancer cells [76].

### 5.3. Sterols

**Stigmasterol**: Stigmasterol solid lipid nanoparticles (Stg-SLNs) were developed for lung cancer therapy, with an evaluation focusing on the cytotoxic effects in lung cancer A549 cells. The results showed strong cytotoxicity and indicated the potential for targeted cancer therapy due to effective drug delivery. The enhanced delivery and efficacy demonstrated by Stg-SLNs suggest their viability as a promising approach for treating lung cancer, leveraging the unique properties of solid lipid nanoparticles for improved therapeutic outcomes [77].

### 5.4. Essential Oils and Complex Mixtures

Essential Oils (*Lippia alba* and *Clinopodium nepeta*): Solid lipid nanoparticles (SLNs) encapsulating essential oils from *Lippia alba* and *Clinopodium nepeta* were developed to enhance the stability and anticancer activity of the oils. The cytotoxic effects and stability of these formulations were evaluated, showing enhanced anticancer activity in lung and colon cancer cells compared to the free essential oils. This enhancement suggests that SLNs provide an effective means of stabilizing and delivering essential oils, thereby increasing their potential as therapeutic agents against cancer [78].

### 5.5. Miscellaneous Natural Compounds

**Prodigiosin**: Prodigiosin, a potent natural red pigment, was entrapped in lyophilized parenteral nanoparticles and rigorously evaluated for its entrapment efficiency, drug release, and in vitro cytotoxicity against various cancer cell lines, including those from triple-negative breast, lung, and colon cancers. Additionally, acute toxicity was studied in rats to assess the safety. The results showed that these nanoparticles effectively inhibited the growth of the cancer cells, suggesting that prodigiosin, when delivered in nanoparticle form, could be a promising therapeutic agent due to its enhanced delivery and potent anticancer properties [79].

Table 3 presents a range of lipid nanoparticle (LNP) formulations designed for the delivery of natural products and their derivatives in cancer therapy. It details each LNP carrier system, the natural active ingredients encapsulated such as curcumin, silymarin, and genistein, along with their therapeutic mechanisms which include enhanced pharmacokinetics, targeted delivery, and synergistic anticancer effects.

## 6. Repurposed Drugs

Repurposing existing drugs for new therapeutic applications offers a cost-effective strategy for expediting lung cancer treatment innovations. Drugs like the antiviral favipiravir [80] and the antibacterial bedaquiline [81] have been investigated for their anticancer potential, facilitated by LNP formulations that optimize their delivery and efficacy.

**Antivirals**: Solid lipid nanoparticles of favipiravir (Fav-SLNs) were evaluated for their drug delivery, cellular uptake, and anticancer effects in the treatment of lung cancer, particularly using nebulized nanoparticles. These nanoparticles induced necrosis and enhanced anti-proliferative properties in lung cancer cells, suggesting a potential new therapeutic use for favipiravir in lung cancer treatment beyond its conventional use for viral infections [80].

**Antibacterials**: Solid lipid nanoparticles loaded with bedaquiline (BQ-SLNs) encapsulated the drug to improve delivery efficiency and alleviate side effects. Evaluations involving particle size, drug release kinetics, and stability were carried out. These nanoparticles exhibited enhanced bioavailability and therapeutic efficacy in the treatment of non-small-cell lung cancer, indicating a promising avenue for optimizing bedaquiline’s utility in cancer therapy [81].

**Antimalarials**: Transitioning from antibacterials to antimalarials, nano-calcium-phosphate-loaded lipid nanoparticles with lumefantrine (LF-CaP-Ls) were assessed for their in vivo anticancer properties in mice models, along with the measurement of 5-methyltetrahydrofolate (5-MTHF) levels in serum. These nanoparticles demonstrated a substantial anticancer effect, reduced tumor progression, and maintained high 5-MTHF levels, indicating their potential as a multifunctional treatment strategy in cancer therapy [82]. Similarly, artemether, traditionally used as an antimalarial agent, was loaded into polyethylene glycol (PEG) solid lipid nanoparticles (ART-SLNs). The development and optimization of these nanoparticles were conducted using a factorial design, focusing on drug loading, entrapment efficiency, and particle size to maximize therapeutic benefits. The evaluation demonstrated high cytotoxicity against lung cancer cells along with a sustained drug release profile, indicating that ART-SLNs might offer a new therapeutic pathway for lung cancer treatment by leveraging the antitumor potential of artemether in a targeted and controlled release formulation [83].

Table 4 showcases lipid nanoparticle (LNP) formulations used for repurposing antiviral and antimalarial drugs as treatments for lung cancer. It details the LNP carrier systems, the repurposed active ingredients like favipiravir and bedaquiline, their mechanisms of action tailored to cancer therapy such as enhancing bioavailability and targeting tumor sites, and references for further validation. The table highlights the innovative use of LNPs to optimize the therapeutic potential and efficacy of these repurposed drugs in combating lung cancer.

## 7. Immunotherapy and Other Treatments

LNPs also play a crucial role in modern immunotherapy strategies, such as delivering immunomodulators like STING agonists [6]. Moreover, pro-apoptotic agents, photosensitizers, and even radiotherapy adjuncts like 5,10,15,20-Tetrakis(4-hydroxy-phenyl)-21H,23H-porphine (Pthpp) loaded onto poly(d,l-lactide-co-glycolide) (PLGA)-lipid hybrid nanoparticles [1] are being redefined by nanotechnology, enhancing their selectivity and effectiveness against lung cancer cells.

**Figure 4:**
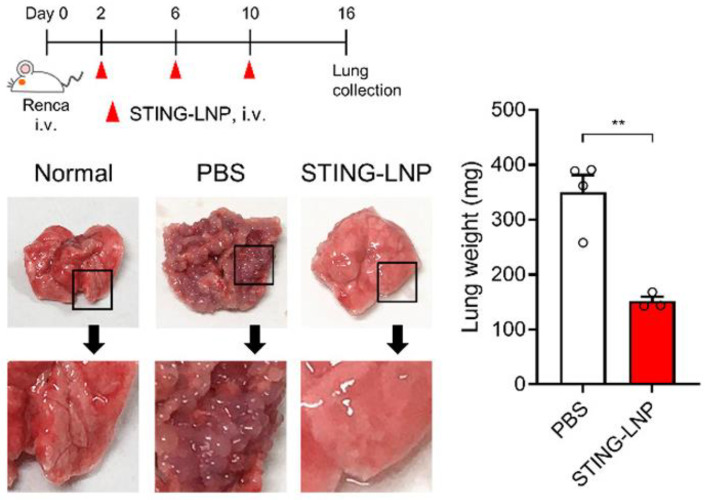
Evaluation of therapeutic effects of STING-LNPs against Renca lung metastasis in vivo: mice with Renca metastasis were intravenously injected three times with the STING-LNPs on days 2, 6, and 10, Mean ± SEM (*n* = 17). The lungs were collected on day 16, Mean + SEM (*n* = 3–4, ** *p* < 0.01) The STING-LNP treatment also significantly reduced the lung weight (152 ± 8 mg) compared with that of the phosphate-buffered saline (PBS)-treated groups (350 ± 31 mg) [6].

**Immunomodulators**: Advancing to immunotherapy, lipid nanoparticles loaded with a stimulator of interferon genes agonist (STING-LNPs) were investigated for their immunotherapy potential against renal tumor lung metastasis in an experimental model, as shown in Figure 4. The evaluation revealed a significant reduction in tumor colonies in lung metastasis, highlighting the effectiveness of STING-LNPs in activating natural killer (NK) cells. This finding suggests that leveraging the innate immune response through STING activation could be a promising strategy for treating metastatic cancer, particularly by enhancing the body’s own immune mechanisms to fight cancer [6].

**Pro-apoptotic Agents**: Further exploring targeted therapies, lipid nanoparticles coated with Apo2L/TRAIL (LUV-TRAIL) were assessed for targeting NSCLC cells resistant to soluble recombinant TRAIL. These evaluations included the cytotoxic effects of LUV-TRAIL on NSCLC cell lines and primary human tumor cells, as well as the synergistic effects when combined with other anti-tumor agents such as flavopiridol and selective inhibitor of cyclin-dependent kinases (CDK) 2, 7, and 9 (SNS-032). The findings indicated that LUV-TRAIL exhibited greater cytotoxic effects and showed enhanced cytotoxicity when used in combination with other anti-tumor agents, underscoring its high clinical potential for treating NSCLC, particularly in resistant forms of the disease [84]. Additionally, solid lipid nanoparticles (SLNs) loaded with B13 (D-NMAPPD), a ceramidase inhibitor, were evaluated for the anticancer activity against lung cancer cells in vitro. The newly synthesized B13-loaded SLNs demonstrated high cytotoxicity against lung cancer cells, suggesting effective anticancer activity. This indicates that targeting specific metabolic pathways in cancer cells, such as ceramide metabolism by inhibiting ceramidase, can be a viable strategy for developing potent anticancer therapies [85].

### Photosensitizers for Photodynamic Therapy (PDT) and Radiotherapy

Exploring advanced therapeutic modalities, poly(d,l-lactide-co-glycolide)-lipid hybrid nanoparticles loaded with 5,10,15,20-Tetrakis(4-hydroxy-phenyl)-21H,23H-porphine (pTHPP) were utilized for photodynamic therapy aimed at overcoming multidrug resistance in lung cancer cells derived from A549 via different resistance mechanisms. These nanoparticles effectively overcame multidrug resistance, showing significant photocytotoxic effects in resistant cell lines. This approach leverages the unique properties of photodynamic therapy to bypass conventional drug resistance mechanisms, offering a new avenue for enhancing treatment efficacy in challenging cancer cases [1]. Additionally, chlorin–lipid nanovesicles encapsulating 131I-labeled bovine serum albumin were designed for a dual therapeutic approach in lung cancer treatment, combining internal radiotherapy with Cerenkov radiation-induced photodynamic therapy (PDT). By utilizing Cerenkov radiation as an internal light source, these nanovesicles generate reactive oxygen species, thereby enhancing the anticancer efficacy. The synergistic effects of radiotherapy and PDT have shown powerful anti-tumor outcomes, significantly prolonging survival and offering a novel and potent option for treating lung cancer [8].

**Nanotechnology-Based Treatments:** Lipid-coated bismuth nanoflowers (DP-BNFs) were prepared for use in combined radiotherapy and photothermal therapy, aimed at treating lung metastatic breast cancer. These nanoflowers enhanced the therapeutic efficiency of both radiotherapy and photothermal therapy, indicating their potential as a multifunctional tool in the treatment of metastatic cancers. The lipid coating contributes to the biocompatibility and effective delivery of the nanoflowers, optimizing their interaction with cancer cells [7].

Table 5 summarizes various lipid nanoparticle (LNP) systems engineered to deliver innovative therapeutic agents for cancer treatment. It includes LNPs loaded with stimulators of interferon genes (STING agonists), TRAIL proteins, and other novel compounds like ceramidase inhibitors and photodynamic therapy agents. Each entry details the carrier system, encapsulated agents, their specific therapeutic actions such as activating immune responses, inducing apoptosis, or enhancing radiosensitivity, and the references for these studies. Table 5 emphasizes the role of LNPs in enabling advanced therapeutic strategies against complex cancers like metastatic renal cell carcinoma and lung cancer.

## 8. Other Therapies

An advanced drug delivery systems like, hyaluronic acid-modified, pH-sensitive lipid-polymer hybrid nanoparticles containing Erlotinib and Bevacizumab (HA-ERL/BEV-LPH NPs) were developed as a dual drug delivery system targeting non-small cell lung cancer (NSCLC). These nanoparticles were evaluated for their particle size, drug release behavior, and tumor inhibition rates in vivo. The studies demonstrated that these nanoparticles significantly reduced tumor volumes and enhanced the accumulation of drugs within the tumor tissues, indicating a potent therapeutic potential for targeting cancer cells more effectively and efficiently [86].

Extending the focus on pulmonary delivery, oly(lactic-co-glycolic acid) porous microspheres loaded with afatinib in stearic acid-based solid lipid nanoparticles and paclitaxel were developed specifically for pulmonary delivery aimed at treating EGFR TKI-resistant non-small cell lung cancer. These microspheres were characterized for their drug release properties, aerodynamics, and in vivo performance in rat models. The system achieved prolonged drug retention in the lungs with minimal systemic exposure, highlighting its potential as a promising treatment option for drug-resistant lung cancer [2].

Further enhancing the strategy for combating resistance, multifunctional lipid-based nanoparticles for the co-delivery of anticancer drugs and siRNA targeting EGFR mutations in NSCLC were developed to contain nanostructured lipid carriers loaded with gefitinib, paclitaxel, and siRNA. This multifunctional delivery system showed enhanced anticancer activity, suggesting potential for improved treatment outcomes in NSCLC with various levels of resistance [87].

Lastly, cabazitaxel-loaded human serum albumin nanoparticles combined with transforming growth factor beta-1 siRNA lipid nanoparticles (CTX-HSA-NPs and TGFβ-1 siRNA LNP) were developed specifically for the treatment of paclitaxel-resistant non-small cell lung cancer. The evaluation covered aspects such as cytotoxicity, tumor inhibition, and drug resistance. These combined therapies enhanced antitumor efficacy and reduced toxicity compared to treatments that did not include TGFβ-1 siRNA, showing promise for overcoming drug resistance and improving patient outcomes in NSCLC treatment [88].

Table 6 details a comprehensive list of lipid nanoparticle (LNP) systems designed for the targeted delivery of chemotherapeutic agents and gene therapy to enhance the treatment of lung cancer and overcome drug resistance. Each entry specifies the LNP carrier system, active ingredients, their combined therapeutic approaches such as synergistic chemotherapy, gene silencing, and targeted delivery mechanisms, along with the corresponding references. The table illustrates cutting-edge developments in nanoparticle technology that improve the efficacy, specificity, and safety in treating non-small cell lung cancer (NSCLC) and other resistant forms of cancer.

## 9. Testing and Evaluation of LNPs in Lung Cancer

### 9.1. Physicochemical Characterization

The foundational characteristics such as particle size and zeta potential are crucial as they impact the LNPs’ biological distribution, cellular uptake, and stability. Particle size can be measured using techniques like dynamic light scattering (DLS) or nanoparticle tracking analysis (NTA), with the optimal range typically being 50–200 nm to exploit the enhanced permeability and retention (EPR) effect for tumor targeting. The zeta potential, indicating the surface charge of LNPs, can be assessed through electrophoretic light scattering, offering insights into their stability and interaction with biological membranes [16,19,26,39].

Another vital parameter is the encapsulation efficiency, which measures how effectively LNPs can encapsulate the therapeutic agent, aiming to maximize therapeutic outcomes while minimizing adverse effects. Techniques such as UV-vis spectroscopy, high-performance liquid chromatography (HPLC), or liquid chromatography–tandem mass spectrometry (LC-MS/MS) are employed to determine the ratio of encapsulated drug to the total drug used in the formulation [18,30,38].

The stability of LNPs is another critical factor, ensuring they maintain their structural integrity and therapeutic capacity under various conditions. Stability testing may include evaluations of physical stability (e.g., aggregation, sedimentation) and chemical stability (e.g., drug degradation), using analytical methods like DLS for monitoring size changes and HPLC for assessing drug degradation [63,78].

For a more comprehensive understanding, advanced physicochemical characterizations are necessary. Techniques such as transmission electron microscopy (TEM) and scanning electron microscopy (SEM) provide intricate details about LNPs’ morphology and structure. Additionally, Fourier transform infrared spectroscopy (FTIR), differential scanning calorimetry (DSC), and X-ray diffraction (XRD) help analyze molecular composition and thermal properties [36,72].

### 9.2. In-Vitro Studies

In lung cancer research, cytotoxicity assays are pivotal for determining the potential toxicity of lipid nanoparticles (LNPs) towards cancer cells, which is essential for optimizing dosage and formulation. The MTT assay is a colorimetric test that assesses cell viability by measuring the reduction of MTT by mitochondrial dehydrogenase to formazan in viable cells. Similarly, the WST-1 assay evaluates cellular metabolic activity and is advantageous due to its water-soluble nature, simplifying handling procedures. The LDH assay, on the other hand, measures the release of lactate dehydrogenase from cells, which occurs when the cell membrane integrity is compromised, thus indicating cytotoxicity [16,19,38].

Understanding how well cells internalize LNPs is crucial for assessing their potential efficacy as drug delivery systems. Techniques such as confocal microscopy provide a detailed visualization of the cellular internalization and intracellular localization of fluorescently labeled LNPs. Flow cytometry quantifies the uptake of LNPs by measuring fluorescence intensity, and fluorescence microscopy is employed to observe and quantify the internalization of these particles [26,39].

Apoptosis assays are fundamental in evaluating whether LNPs can induce programmed cell death in cancer cells, an important mechanism for anticancer therapies. The annexin V/PI staining method, utilized via flow cytometry, differentiates between early apoptotic, late apoptotic, and necrotic cells. Caspase activity assays measure the activation of caspases, key mediators of apoptosis, while the TUNEL assay detects DNA fragmentation, a hallmark of apoptosis [15].

For LNPs designed for gene therapy applications, assessing transfection efficiency is critical. The luciferase reporter assay is used to evaluate the activity of a reporter gene, which indicates the successful delivery and expression of DNA. GFP expression monitoring serves as a marker to visualize and quantify transfection efficiency. Quantitative PCR (qPCR) is also utilized to measure the levels of specific DNA or RNA molecules, providing insights into expression levels post-transfection [30].

### 9.3. In-Vivo Studies

Evaluating the anti-tumor efficacy of lipid nanoparticles (LNPs) is a critical aspect of cancer research, particularly in the context of lung cancer. To assess the effectiveness of LNPs in inhibiting tumor growth, it is essential to utilize relevant animal models. Researchers administer LNPs to these models and monitor tumor progression using various methods, such as caliper measurements for externally palpable tumors or advanced imaging techniques for internal tumors [17,20].

Understanding the biodistribution of LNPs is crucial for evaluating their targeting capabilities and overall distribution within the body. This involves labeling LNPs with radioactive isotopes or fluorescent tags and tracking their localization over time using techniques like gamma scintigraphy or fluorescence imaging. Such studies are pivotal for optimizing the nanoparticle design to enhance target specificity and minimize off-target effects [19,24].

Systemic toxicity studies are vital to establish the safety of LNPs. These studies typically use chronic or acute exposure models, where animals are administered various doses of LNPs. Key observations include monitoring behavioral changes, weight, and conducting histopathological examinations of major organs post-mortem to evaluate any toxic effects. Additionally, blood biochemistry and hematology analyses are integral to these assessments [21,31].

The pharmacokinetics of LNPs, including their absorption, distribution, metabolism, and excretion (ADME) properties, are studied to understand key pharmacological attributes. Techniques such as high-performance liquid chromatography (HPLC), liquid chromatography–tandem mass spectrometry (LC-MS/MS), or radioactivity measurements are employed to measure the concentrations of LNPs or their loaded drugs in blood and other tissues at various time points. These investigations are crucial for determining the circulation time, metabolic stability, and excretion routes [24,37].

## 10. Status of Current Clinical Trials

The future direction of lipid nanoparticle (LNP) technologies in lung cancer treatment is poised for significant advancements through rigorous clinical trials and innovations in regulatory frameworks. These efforts are crucial for validating the efficacy and safety of LNPs and ensuring their effective application across diverse patient demographics and lung cancer subtypes. The development of streamlined regulatory pathways will be instrumental in accelerating the testing and approval of these novel nanoparticle formulations, ultimately fostering their clinical adoption.

Recent investigations have highlighted the potential of the intravenous administration of tumor mRNA-loaded lipid particles (LPs) in targeting lung cancers. These RNA-LPs have been shown to localize primarily to the lung, where they transfect antigen-presenting cells and trigger an activated T-cell response, inducing anti-tumor immunity. This mechanism is distinct from other formulations as RNA-LPs engage multiple arms of the immune system, both innate and adaptive, and significantly remodel the systemic and intratumoral immune milieu [95]. This comprehensive immune response is particularly important as it addresses the potent barriers that currently limit the efficacy of vaccine-, cellular-, and checkpoint-inhibiting immunotherapies.

Further validating the promise of LNPs in clinical settings, several ongoing trials are exploring their potential. For example, the study of quaratusugene ozeplasmid (Reqorsa), combined with pembrolizumab in previously treated non-small cell lung cancer (NSCLC), was structured to determine optimal dosing and evaluate safety and efficacy through a two-phased approach [96]. Similarly, the combination of quaratusugene ozeplasmid with atezolizumab as maintenance therapy for patients with extensive stage small cell lung cancer (ES-SCLC) is under evaluation, with specific attention to toxicities and dose-limiting toxicities assessed according to established criteria [97].

In addition, quaratusugene ozeplasmid is being investigated in combination with osimertinib in NSCLC patients with activating EGFR mutations who have progressed on osimertinib treatment. This trial aims to determine the safety and efficacy of this novel systemic gene therapy approach in a phased clinical trial setting [98].

These clinical trials represent a significant step forward in the application of LNPs in cancer therapy. By systematically evaluating these innovative treatments in diverse lung cancer settings, researchers hope to overcome current therapeutic limitations and offer new, effective options for patients battling this challenging disease.

## 11. Collective Achievements

**Enhanced Drug Delivery and Efficacy:** LNPs have significantly improved the delivery and efficacy of chemotherapy drugs, achieving high encapsulation efficiency and targeted release. Notable formulations include multi-trigger responsive nanovesicles encapsulating paclitaxel [15], and hybrid nanoparticles [16,17,19,21,22,23]. These LNPs have been tailored for targeted delivery, significantly reducing tumor size and enhancing survival rates.

**Targeted Drug Delivery:** LNPs have demonstrated success in targeted delivery to lung cancer cells, enhancing therapeutic effectiveness. Key examples include mannosylated, chitosan-coated, and transferrin-conjugated systems [24,26,30].

**Overcoming Drug Resistance:** LNPs such as paclitaxel-encapsulated PLGA–lipid hybrids and other innovative designs have targeted specific cancer cell characteristics and mechanisms, enhancing antitumor ability and overcoming resistance [21,27,35,37,48,50,52,71].

**Reduced Toxicity and Side Effects:** The strategic formulation of LNPs, such as EGF conjugated hybrids and PEGylated nanostructured carriers, have shown reduced systemic toxicity and enhanced tumor-specific activity [20,23,30,33,38,39,88,92].

**Enhanced Efficacy with Specificity:** LNPs like biotin-decorated sunitinib-loaded carriers and transferrin-modified systems have increased drug delivery specificity and cellular uptake, significantly improving therapeutic efficacy [5,9,41,47,49,57,73,74,75,83,84,89].

**Controlled Release and Sustained Efficacy:** LNPs such as crizotinib-loaded and pazopanib-loaded carriers provide controlled and sustained drug release, enhancing therapeutic effectiveness while minimizing side effects [36,40,82].

**Combination Therapy and Synergistic Effects:** LNPs have facilitated the co-delivery of multiple therapeutic agents, enhancing their synergistic therapeutic effects. This includes lipid–polymer hybrids co-delivering erlotinib and bevacizumab, and other combinations like doxorubicin and paclitaxel [4,8,65,68,86,90].

**Specialized Drug Release Mechanisms:** LNPs designed with specific release profiles, such as pH-sensitive mechanisms, optimize the therapeutic window and enhance anticancer effects, exemplified by lumefantrine and nano-calcium phosphate-loaded LNPs [82].

## 12. Limitations

**Complexity of Design and Manufacture:** LNPs require sophisticated design and precise control over particle size, surface characteristics, and drug release profiles. These complexities pose challenges in scaling up production and ensuring batch-to-batch consistency, crucial for clinical applications. The advanced engineering required can be a barrier to scaling up for commercial production while maintaining consistency and stability during complex manufacturing processes.

**Biocompatibility and Safety Issues:** Despite advancements, there are significant concerns about the long-term biocompatibility and potential immune responses of LNPs. Ensuring the safety of LNPs over extended periods and across diverse patient populations is critical, with ongoing needs for comprehensive studies to address these issues thoroughly.

**Regulatory and Commercialization Hurdles:** The pathway from laboratory research to market involves rigorous regulatory scrutiny, which can be lengthy and costly. Demonstrating consistent, reproducible benefits in clinical settings is challenging, complicating rapid development and deployment. LNPs face rigorous regulatory scrutiny due to their novel composition and mechanisms of action, which can delay their approval and clinical adoption.

**Variability in Clinical Outcomes:** Despite promising preclinical results, translating these outcomes to clinical settings often results in variable efficacy due to biological complexities and individual patient variability. These inconsistencies can affect the generalizability of the results and the predictability of treatment outcomes, complicating the translation of LNPs into effective treatments.

## 13. Future Directions

**Enhanced Targeting Capabilities:** Future research will focus on refining the targeting capabilities of LNPs using sophisticated ligands or stimuli-responsive systems to more accurately target tumor cells while sparing normal tissues, enhancing delivery efficiency and reducing off-target effects [15,21].

**Multi-drug and Combinatorial Therapies:** Developing LNPs that can deliver multiple drugs simultaneously to exploit synergistic effects will enhance treatment efficacy and help in overcoming resistance mechanisms. This includes expanding the use of LNPs to deliver combination therapies that can simultaneously target multiple pathways or mechanisms in cancer cells [17,21,29,47,51,58].

**Personalized Medicine:** Tailoring LNP formulations to the genetic and molecular profile of individual tumors will advance personalized medicine, offering treatments that are more effective and have fewer side effects. This requires integrating bioinformatics, genomic data, and patient-specific responses into the nanoparticle design process.

**Advanced Manufacturing Techniques:** Innovations in manufacturing, such as automation and AI-driven systems, are necessary to address current limitations in scalability and reproducibility. Developing more robust and scalable manufacturing processes will ensure that LNPs can be produced consistently and cost-effectively.

**Clinical Trials and Regulatory Innovation:** Conducting more comprehensive clinical trials and long-term studies will be crucial for validating the efficacy and safety of LNPs, ensuring their suitability across different populations and subtypes of lung cancer. Streamlined regulatory pathways that facilitate the testing and approval of novel nanoparticle formulations will accelerate their clinical adoption.

**Interdisciplinary Collaboration:** Ongoing collaboration across multiple disciplines, integrating insights from material science, bioinformatics, pharmacology, and oncology, is essential to tackle the multifaceted challenges of LNP development and to innovate solutions tailored to lung cancer treatment.

**Integration with Emerging Therapies:** Integrating LNPs with emerging therapies such as gene editing tools and immunotherapies could open new avenues for comprehensive cancer treatment strategies, targeting various aspects of cancer biology.

## 14. Conclusions

In the pursuit of enhancing lung cancer treatment, lipid-based nanoparticles (LNPs) have demonstrated remarkable achievements, offering targeted drug delivery, overcoming resistance, and reducing toxicity. However, their realization faces formidable limitations, including complex manufacturing, biocompatibility concerns, regulatory hurdles, and variability in clinical outcomes. Looking forward, the future of LNPs in lung cancer therapy lies in refining targeting capabilities, embracing multi-drug therapies, advancing personalized medicine, and innovating manufacturing processes. Moreover, comprehensive clinical trials and regulatory innovation are imperative for validating efficacy and safety. Interdisciplinary collaboration and integration with emerging therapies hold the promise of revolutionizing lung cancer treatment. By addressing these challenges collectively, LNPs can evolve into a transformative tool in the fight against lung cancer, offering hope for improved outcomes and quality of life for patients.

## Figures and Tables

**Table 1:** Overview of lipid-based nanocarriers for cancer chemotherapy.

Lipid Nanoparticles (LNP) Carrier System	Active Ingredient(s) Encapsulated	Mechanism of Therapy	Ref.
Pulmonary surfactant mimetic lipid vesicles	Paclitaxel	Chemotherapy	[15]
Poly(lactic-co-glycolic acid) (PLGA)–Lipid Hybrid	Paclitaxel	Chemotherapy	[16]
Nanostructured lipid carriers (NLC)	Paclitaxel and Doxorubicin	Combination Chemotherapy	[17]
Lipid-based nanoemulsion	Docetaxel	Chemotherapy	[18]
Nanostructured lipid carriers (NLC)	Celecoxib and Docetaxel	Chemotherapy and Anti-inflammatory	[19]
Epidermal Growth Factor-conjugated lipid–polymer hybrid nanoparticles	Docetaxel, Resveratrol	Targeted Chemotherapy	[20]
Transferrin-decorated protein–lipid hybrid nanoparticles	Cisplatin, Docetaxel	Targeted Chemotherapy	[21]
Lipid–polymer hybrid nanoparticles with polyvinyl alcohol	Etoposide	Controlled Drug Delivery	[22]
Polyethylene glycol-modified nanostructured lipid carriers	10-Hydroxycamptothecin	Enhanced Anti-tumor Effect	[23]
Mannosylated solid lipid nanoparticles	Gemcitabine	Targeted Chemotherapy	[24]
Chitosan-coated solid lipid nanoparticles	Methotrexate	Interventional Delivery for Lung Cancer	[26]
Ultra-small lipid–polymer nanoparticles	Cis-Platinum	Targeted Chemotherapy for Lung Cancer	[27]
Post-bombesin- and pre-bombesin-decorated nanostructured lipid carriers	Doxorubicin and Deoxyribonucleic Acid	Targeted Combination Therapy for Lung Cancer	[29]
Transferrin-conjugated lipid-coated poly(d,l-lactic-co-glycolic acid) nanoparticles	Doxorubicin	Targeted Therapy for Lung Cancer	[30]
Core-shell-type lipid–polymer hybrid nanoparticles composed of hydrogenated soy phosphatidylcholine and 1,2-distearoyl-sn-glycero-3-phosphoethanolamine-*N*-[methoxy(polyethylene glycol)-2000]	Erlotinib	Targeted Delivery for Non-Small Cell Lung Cancer	[31]
Compritol/Poloxamer 407	Erlotinib	Targeted Therapy for Non-Small Cell Lung Cancer	[32]
Polyethylene glycol (PEG)-modified polypeptide lipid nano capsules	Erlotinib	Enhanced Anticancer Efficacy for Non-Small Cell Lung Cancer	[33]
Nanostructured lipid carriers and liposomes	Erlotinib	Comparative Oncologic Effectiveness against A549 Cell Line	[34]
Lipid-core nanocapsules	Tretinoin	Overcoming Resistance and Enhancing Delivery to Lung Cancer Cells	[35]
Solid lipid nanoparticles	Pazopanib	Enhanced Oral Bioavailability and Therapeutic Efficacy against Non-Small Cell Lung Cancer	[36]
Transferrin-modified, redox-sensitive lipid–polymer hybrid nanoparticles	Afatinib	Targeted, Redox-Sensitive Delivery for Non-Small Cell Lung Cancer	[37]
Nanostructured lipid carrier	Gefitinib	Lymphatic Drug Delivery for Metastatic Lung Cancer Treatment	[38]
Biodegradable lipid nanoparticles (NLC)	Gefitinib and Azacitidine	Co-delivery for Treatment of Metastatic-Resistant Lung Cancer	[39]
Lipid–Polymer Hybrid Nanoparticles using Poly(lactic-co-glycolic acid) (PLGA), Poly(caprolactone) (PCL), Soy Phosphatidylcholine, and 1,2-Distearoyl-sn-glycero-3-phosphoethanolamine-*N*-[methoxy(polyethylene glycol)] (DSPE-PEG)	Crizotinib	Targeted Chemotherapy for Non-Small Cell Lung Cancer	[40]
Biotin-decorated nanostructured lipid carriers	Sunitinib	Targeted Chemotherapy for Lung Cancer	[41]
Liposomes with cationic polar lipid 1,2-dioleoyl-3-trimethylammonium-propane (DOTAP) and Cholesterol	All-trans Retinoic Acid	pH-responsive Molecular Therapy for Lung Cancer	[42]
Hybrid lipid nanocore–protein shell composites with mannitol, hydroxypropyl-beta-cyclodextrin, and leucine	Genistein and All-Trans Retinoic Acid	Combined Delivery for Targeted Lung Cancer Therapy	[43]
Solid lipid nanoparticles	Phospho-sulindac (OXT-328)	Mitochondrial Targeting and Enhanced Antitumor Efficacy	[44]
Chitosan nanoparticles modified with polyethylene glycol, epidermal growth factor receptor-binding peptide, and lipid derivatives	Lipid-modified Cisplatin	Targeted Chemotherapy with Enhanced Cellular Cytotoxicity	[45]

**Table 2:** Lipid-based nanoparticles for gene and drug delivery in cancer therapy.

LNP Carrier System	Active Ingredient(s) Encapsulated	Mechanism of Therapy	Ref.
Cationic solid lipid nanoparticles composed of tricaprin, 3β-[*N*-(*N*′,*N*′-dimethyl aminoethane) carbamoyl] cholesterol, dioleoyl phosphatidylethanolamine, and Tween 80	Plasmid DNA encoding p53 gene (pp53-EGFP)	Gene Therapy to Induce Apoptosis and Tumor Growth Inhibition	[46]
Solid lipid nanoparticles with Transferrin (Tf) containing ligands	Doxorubicin and Enhanced Green Fluorescence Protein Plasmid (pEGFP)	Combination of Gene Therapy and Chemotherapy for Lung Cancer	[47]
pH-Sensitive Lipid (YSK05), GALA peptide	Small interfering RNA (siRNA)	Gene Silencing and Regression of Metastatic Lung Cancer	[48]
Nanostructured lipid carriers with Transferrin (Tf) containing ligands	Enhanced Green Fluorescence Protein Plasmid (pEGFP)	Gene Therapy for Lung Cancer	[49]
Cationic solid lipid nanoparticles	Plasmid DNA for STAT3 RNA interference	Downregulation of STAT3 to overcome chemotherapy resistance	[50]
Tripeptide lipid, sucrose laurate, and folate-polyethylene glycol-di-stearoyl-phosphatidylethanolamine	Paclitaxel and Vascular endothelial growth factor (VEGF) small interfering RNA	Co-delivery of chemotherapy and gene silencing for enhanced tumor inhibition	[51]
Self-assembled lipid nanoparticles	Cisplatin prodrug and siRNA targeting xeroderma pigmentosum group F (XPF)	Ratiometric co-delivery to combat drug resistance by enhancing DNA damage and inhibiting repair	[52]
Polyethylene glycol cholesterol and tocopherol polyethylene glycol 1000 succinate-modified lipid nanoparticles	Transforming Growth Factor beta1 small interfering RNA	Gene silencing to treat paclitaxel-resistant non-small cell lung cancer	[53]
Liposome prepared using lipid film-coated proliposomes	CYP1A1 small interfering RNA	Gene silencing to inhibit lung cancer growth linked to polycyclic aromatic hydrocarbons	[54]
Proprietary lipid nanoparticle (LNP)	Small interfering RNA (siRNA) against Glutathione S-transferase Pi (GSTP)	RNA interference to downregulate GSTP and inhibit oncogenic pathways	[55]
Transferrin (Tf)-decorated nanostructured lipid carriers	Paclitaxel and Enhanced Green Fluorescence Protein Plasmid (pEGFP)	Co-delivery of Anticancer Drug and DNA for Lung Cancer Therapy	[57]
Solid lipid nanoparticles	Anti-microRNA oligonucleotides (AMOs) targeting microRNA-21	Suppression of microRNA-21 to decrease tumor cell proliferation, migration, and invasion	[58]
Lipid nanoparticles composed of quaternary amine-tertiary amine cationic lipid combination (QTsome)	AntimiR-21 (AM-21) oligonucleotide	Therapeutic delivery of AntimiR-21 to inhibit gene silencing activities of miR-21 in lung cancer	[59]
Coated cationic lipid-nanoparticles entrapping miR-660	microRNA-660 (miR-660)	Replacement therapy to inhibit lung cancer growth by modulating the MDM2-P53 axis	[60]
1,2-dioleoyl-3-trimethylammonium-propane (DOTAP)/Egg Phosphatidylcholine (PC)/Cholesterol/Tween 80	G3139-GAP (Antisense Oligonucleotide Gapmer Against Bcl-2)	Antisense oligonucleotide therapy to downregulate Bcl-2 expression	[61]

**Table 3:** Lipid nanoparticle delivery of natural products for cancer therapy.

LNP Carrier System	Active Ingredient(s) Encapsulated	Mechanism of Therapy	Ref.
Catanionic lipid nano system (CLNs)	Curcumin (CCM)	Enhanced pharmacokinetics and anticancer activity	[62]
Solid Lipid Nanoparticles (SLNs)	Curcumin (Cur-SLNs)	Sustained release and enhanced cellular uptake	[63]
Solid Lipid Nanoparticles (SLNs)	Curcumin	Enhanced bioavailability and targeting to lung cancer	[64]
Solid Lipid Nanoparticles (SLNs)	Paclitaxel and Curcumin (PC-SLNs)	Synergistic oncotherapy and enhanced drug delivery	[65]
Nanostructured Lipid Carriers (NLCs) with folate (FA) appendage	Docetaxel and Curcumin (FA-DTCR-NLCs)	Targeted, synergistic combination oncotherapy	[66]
Anisamide-lipid-coated calcium phosphate nanoparticles	Oridonin (ORD)	Targeted delivery and enhanced anti-tumor efficiency in lung cancer	[67]
Layer-by-layer nanoparticles	Cisplatin (Cis-diamine dichloroplatinum) and Oridonin	Synergistic combination therapy for enhanced antitumor activity	[68]
Multicompartmental lipid–protein nanohybrids (MLPNs)	Tretinoin (TRE) and Genistein (GEN)	Combined delivery for synergistic anti-lung cancer therapy	[69]
Solid Lipid Nanoparticles (SLNs)	Silymarin	Enhanced cytotoxic and apoptotic effects on lung and breast cancer cells	[70]
Hyaluronic acid-based nanostructured lipid carriers (NLCs)	Apigenin (APG) and Docetaxel (DTX)	Nrf2-dependent apoptosis induction in lung cancer cells	[71]
Solid Lipid Nanoparticles (SLNs)	Phyllanthi Tannin (PTF)	Enhanced anti-tumor efficacy and safety for lung cancer therapy	[72]
Nanostructured Lipid Carriers (NLCs)	Geraniol	Improved bioavailability and enhanced anticancer activity	[73]
Lipid–polymer hybrid nanoparticles	Paclitaxel and Triptolide	Synergistic combination therapy to reduce drug resistance	[74]
pH-sensitive nanostructured lipid carriers	Doxorubicin and Beta-Elemene	Co-delivery for synergistic anticancer effect with pH sensitivity	[75]
Solid lipid nanoparticles	Sclareol	Antiproliferative effects via genotoxicity and cytotoxicity	[76]
Solid Lipid Nanoparticles (Stigmasterol-based)	Stigmasterol	Induces caspase-3/7 activation, operates through oxidative stress, necrosis independent	[77]
Solid Lipid Nanoparticles	*Lippia alba* and *Clinopodium nepeta* Essential Oils	Enhanced anticancer activity, increased cytotoxicity in lung and colon cancer cells	[78]
Lipid–Polymer Hybrid Nanoparticles	Prodigiosin	Enhanced bioactivity against cancer cells, high entrapment efficiency, promotes cellular uptake	[79]

**Table 4:** Repurposed drugs delivered via lipid nanoparticles for lung cancer therapy.

LNP Carrier System	Active Ingredient(s) Encapsulated	Mechanism of Therapy	Ref.
Solid Lipid Nanoparticles	Favipiravir	Improves drug delivery to lung cancer sites, enhances anti-proliferative properties	[80]
Solid Lipid Nanoparticles	Bedaquiline	Enhances biodistribution and bioavailability, reduces tumor volume and weight effectively in NSCLC	[81]
Nano calcium phosphate-loaded lipid nanoparticles (LF-CaP-Ls)	Lumefantrine	pH-sensitive mechanism, improved 5-methyltetrahydrofolate levels, reduced tumor weight	[82]
PEGylated solid lipid nanoparticles	Artemether	Downregulates matrix metalloproteinase (MMP-9), hypoxia-inducible factor (HIF-1a), Vascular endothelial growth factor (VEGF); cytotoxic effect on tumor cells	[83]

**Table 5:** Lipid Nanoparticles for Innovative Cancer Therapies.

LNP Carrier System	Active Ingredient(s) Encapsulated	Mechanism of Therapy	Ref.
Poly(d,l-lactide-co-glycolide)-lipid hybrid nanoparticles	5,10,15,20-Tetrakis(4-hydroxy-phenyl)-21*H*,23*H*-porphine (pTHPP)	Photodynamic therapy (PDT) to overcome multidrug resistance and metastasis-associated MDR in lung cancer	[1]
Lipid nanoparticles loaded with a STING agonist	Stimulator of Interferon Genes Agonist	Activates natural killer cells, induces antitumor effect against metastatic renal cell carcinoma	[6]
Lipid-coated bismuth nanoflowers	None specified (thermos-radio sensitizer)	Thermo-radiotherapy for lung metastatic breast cancer	[7]
Chlorin-lipid nanovesicle, 131I-labeled bovine serum albumin	Chlorin e6 (Ce6), 131I	Synergistic radiotherapy and Cerenkov radiation-induced photodynamic therapy	[8]
Lipid nanoparticles coated with TRAIL	Apo2-Ligand/TRAIL (tumor necrosis factor-related apoptosis-inducing ligand)	Induces apoptosis in tumor cells, overcomes resistance to soluble recombinant TRAIL	[84]
Solid lipid nanoparticles	B13 (D-NMAPPD, a ceramidase inhibitor)	Enhances anticancer activity with high cytotoxicity on lung cancer cells	[85]

**Table 6:** Other lipid nanoparticle systems for targeted cancer therapy.

LNP Carrier System	Active Ingredient(s) Encapsulated	Mechanism of Therapy	Ref.
Stearic acid-based solid lipid nanoparticles and poly-lactide-co-glycolide-based porous microspheres	Afatinib, Paclitaxel	Chemotherapy for EGFR TKIs resistant non-small cell lung cancer (NSCLC)	[2]
Nanostructured lipid carriers with *N*-acetyl-d-glucosamine, poly(6-*O*-methacryloyl-d-galactopyranose)-Gemcitabine/Paclitaxel conjugates	Gemcitabine, Paclitaxel	Combination chemotherapy, targeted therapy	[3]
Lipid-coated hollow calcium phosphate nanoparticles	Doxorubicin, Paclitaxel	Synergistic co-delivery, chemotherapy	[4]
Aptamer-conjugated lipid-polymer hybrid nanoparticles, redox-sensitive docetaxel prodrug	Docetaxel prodrug, Cisplatin	Targeted chemotherapy, overcoming drug resistance in NSCLC	[5]
Hyaluronic acid-modified, pH sensitive lipid–poly(lactic-co-glycolic acid) nanoparticles with CD133 aptamers	All-trans Retinoic Acid	Targeted therapy, differentiation	[9]
Lipid nanocapsules containing a lauroyl derivative of gemcitabine	Gemcitabine derivative	Targeting lymph nodes and combating mediastinal metastases in non-small cell lung cancer	[25]
RGD peptide-modified, redox-sensitive lipid–polymer nanoparticles	Paclitaxel prodrug, Cisplatin	Targeted, synergistic chemotherapy for enhanced lung cancer treatment	[28]
Hyaluronic acid-modified, pH sensitive lipid–polymer hybrid nanoparticles with adipic acid dihydrazide	Erlotinib, Bevacizumab	Targeted delivery, chemotherapy, angiogenesis inhibition	[86]
Nanostructured Lipid Carriers, luteinizing hormone release hormone, EGF-TK inhibitor, gefitinib, paclitaxel, siRNA, and rhodamine	Gefitinib, Paclitaxel, siRNA	Targeted chemo and gene therapy, imaging for EGFR mutations and resistance	[87]
Human serum albumin nanoparticles combined with TGFβ-1 siRNA lipid nanoparticles	Cabazitaxel, TGFβ-1 siRNA	Combined chemo- and gene therapy for paclitaxel-resistant non-small cell lung cancer (NSCLC)	[88]
CD133+ targeting peptide TISWPPR, PEG-modified nanostructured lipid carriers	Paclitaxel, Salinomycin	Chemotherapy, cancer stem cell targeting	[89]
Oleic acid-based solid lipid nanoparticles	Gemcitabine, Oxaliplatin	Combined chemotherapy	[90]
Nanostructured lipid carriers co-loaded with etoposide prodrug (linked to polyethylene glycol) and cisplatin	Etoposide Prodrug, Cisplatin	Chemotherapy	[91]
Stealth polymer–lipid hybrid nanoparticles co-loaded with doxorubicin and mitomycin C	Doxorubicin, Mitomycin C	Combination chemotherapy	[92]
Cetuximab-functionalized nanostructured lipid carriers for the co-delivery of paclitaxel and 5-Demethylnobiletin	Paclitaxel, 5-Demethylnobiletin	Targeted combination therapy	[93]
Solid lipid core nanocapsules with a PEGylated polymeric corona, loaded with paclitaxel and erlotinib	Paclitaxel, Erlotinib	Combination chemotherapy	[94]

## Data Availability

Data sharing is not applicable to this article.
